# Timing of rapid weight gain and its effect on subsequent overweight or obesity in childhood: findings from a longitudinal birth cohort study

**DOI:** 10.1186/s12887-020-02184-9

**Published:** 2020-06-12

**Authors:** Yi-Fan Li, Shio-Jean Lin, Tung-liang Chiang

**Affiliations:** 1 Division of Clinical Chinese Medicine, National Research Institute of Chinese Medicine, Ministry of Health and Welfare in Taiwan, Taipei, Taiwan; 2 Department of Pediatrics, Chi Mei Medical Center, Taipei, Taiwan; 3 Institute of Health Policy and Management, College of Public Health, National Taiwan University, Room 620, No. 17, Xu-Zhou Road, Taipei, Taiwan 10055 Taiwan

**Keywords:** Children, Rapid weight gain, Overweight, Obesity

## Abstract

**Background:**

Rapid weight gain (RWG) has been recognized as an important determinant of childhood obesity. This study aims to explore the RWG distribution among children at six-month intervals from birth to two years old and to examine the association of RWG in each interval with overweight or obesity development in preschool- and school-aged children.

**Methods:**

Data were obtained from the Taiwan Birth Cohort Study, which is a nationally representative sample of 24,200 children who participated in a face-to-face survey. A total of 17,002 children had complete data both for weight and height at each of the five measurement time periods. Multivariable logistic regression models quantified the relationship between RWG and childhood overweight or obesity.

**Results:**

A total of 17.5% of children experienced rapid weight gain in the first six months of age, compared to only 1.8% of children from 18-24 months. RWG was significantly associated with an increased risk of developing overweight or obesity at 36 months (RWG birth-6 months: OR = 2.6, 95% CI: 2.3–2.8; RWG 18–24 months: OR = 3.7, 95% CI: 2.9–4.6), 66 months (RWG birth-6 months: OR = 2.2, 95% CI: 2.0–2.4; RWG 18–24 months: OR = 2.3, 95% CI: 1.8–2.8), and 8 years of age (RWG birth-6 months: OR = 1.7, 95% CI: 1.6–1.9; RWG 18–24 months: OR = 2.4, 95% CI: 2.0–3.0).

**Conclusions:**

Childhood RWG increased the risk of subsequent overweight or obesity, regardless of the specific time interval at which RWG occurred before the age of two years. The results reinforce the importance of monitoring childhood RWG continuously and show the risks of childhood RWG with respect to the development of overweight or obesity at preschool and school ages.

## Background

Childhood obesity continues to be a critical public health problem worldwide. The global prevalence of childhood obesity increased dramatically from 1.0% in 1975 to 9.0% in 2016 [1]. A growing body of evidence indicates that childhood obesity increases the risk of obesity in adolescence and adulthood and the incidence of noncommunicable diseases (NCDs), such as cardiovascular diseases, cancers, and diabetes, later in life [2, 3]. From the life-course perspective, addressing childhood obesity is critical for the prevention and control of NCDs [4].

Fast postnatal weight accumulation, or rapid weight gain (RWG), has been recognized as an important determinant of childhood obesity [5–8]. Two systematic reviews by Ong and Loos [7] and Zheng et al. [8] reported that children with RWG before the age of two were more likely to become overweight/obese than children without RWG, with adjusted odds ratios from 1.4 to 6.8. Accordingly, professional organizations such as the American Academy of Pediatrics (AAP) [9] and the Institute of Health Visiting in the UK [10] have recognized that early RWG in children should be targeted to prevent childhood overweight and obesity and have therefore recommended that parents and health care providers observe children’s growth patterns starting at birth.

However, evidence regarding the timing of RWG for intervention to promote health is inconsistent. A body of research has explored the association of various timings of RWG with health outcomes such as obesity and cardiovascular diseases. These studies reported that the critical timing of RWG occurred during early childhood in the first six months [11, 12], first 12 months [13, 14], or first 24 months of life [15–17]. Other studies reported that the important RWG occurred during early infancy, including the first week [18] or the first four months of life [19]. The diverse results of RWG timing in children might be due to various limitations of the study designs. For example, there was a lack of evidence from a large sample size and a longitudinal cohort study to collect anthropometric data at regular intervals after birth [20]. In addition, little is known about the pattern of RWG according to time in early life. It is unclear which specific period of childhood is critical for RWG and whether the occurrence of RWG follows a specific pattern.

Therefore, the current study, which uses data from the Taiwan Birth Cohort Study (TBCS), aims first to describe the distribution of childhood RWG from birth to 24 months of age and second to examine the various effects of RWG occurring during different periods before the age of two on the development of childhood overweight or obesity in preschool (age 36 months and 66 months) and school age (age eight) children.

## Methods

This study has been approved by the Research Ethics Committee of National Taiwan University (NTU-REC) on March 25, 2019 (NTU-REC No: 201902HM003).

### Study design and setting

This study was based on data from the TBCS, which is the first large-scale, longitudinal design and was supported financially and administratively by the Health Promotion Administration (HPA), Ministry of Health and Welfare in Taiwan. By following nationally representative children from birth through young adulthood, the TBCS aims to record and evaluate child health, explore social determinants of child health, and investigate the early origins of adult health based on the child’s life course. Therefore, TBCS collected a wide range of information at various stage in life pertaining to each child’s health and development, lifestyle, parenting, childcare, and social environment. The current study used panel data to describe the distribution of RWG during the growth period and to further explore the association of RWG before 24 months of age with overweight and obesity at preschool and school age.

### Participants

The TBCS enrolled 24,200 infants born throughout the year in 2005 who were initially selected from 206,741 live births based on the National Birth Report Database by using two-stage stratified random sampling. Initially, primary sampling units (PSUs) were townships identified geographically in Taiwan. A total of 85 PSUs were sampled randomly according to 12 levels stratified based on urbanization and the total fertility rate of townships in sequence. Second, a total of 24,200 individuals were selected from the PSUs by simple random sampling determined by probability proportionate to size (PPS) and the order of each birth month. Overall, the average sampling rate was approximately 11.7%. A total of 21,248 (87.8%) children completed the baseline survey at the age of six months and were recruited as cohort members from the 24,200 eligible children. Follow-up interview surveys were subsequently conducted at 18 months, 3 years, 5 years, and 8 years of age, with response rates of 94.9, 93.7, 92.8, and 91.9%, respectively. The present study sample included 17,002 (80.2%) children after excluding those who experienced RWG after the age of two (n = 1464) from among the children who completed all four waves of the follow-up surveys (n = 18,466).

All participants received a letter before each survey wave from the HPA, with information about TBCS, including its purposes, research methods, confidential process, and contact information of the administrator. The interviews were initiated after the children’s parents or guardians understood their rights and completed the informed consent form.

### Measures

Each wave of TBCS survey was conducted via face-to-face interviews using structural questionnaires answered by either the mother or a primary caregiver. Four steps were followed to develop a TBCS questionnaire. First, the conceptual framework and study plans according to the objectives of TBCS were developed by the principal investigator, co-principal investigators and staff from the HPA. Second, the questionnaire was constructed with reference to previous research and social contexts before each wave of survey. Third, participants’ comments and feedback were collected to revise the questionnaires after the implementation of the pretest and pilot study. Finally, the protocol and questionnaires of the TBCS were approved by the Directorate-General of Budget, Accounting and Statistics in the Executive Yuan, according to the Statistics Act of Taiwan.

#### Anthropometric data

Children’s physical growth data in TBCS were primarily obtained from parents based on the structural questionnaires, included questions regarding anthropometric data, date of measurement, and data sources. Before each wave of the TBCS survey, an official letter was sent to each cohort member’s parents, reminding them to prepare the children’s anthropometric data. The data provided by the parents came from two sources. The first source is the Children’s Health Booklet, which parents or primary caregivers prepared for interviews with the TBCS. The Children’s Health Booklet records children’s health status and primary health care information, including anthropometric data and compulsory vaccination records, based on seven free well-childcare visits under the National Health Insurance guidelines in Taiwan. Health care providers measure and record children’s length/height, weight, and head circumference during each well-child care visit. The second source is parental reports including measurements performed by the parents or obtained from kindergarten. Based on our previous study, we found that 80% of the anthropometric data in TBCS before age three were from well-child visits, and 60% of data after age three were from parents’ measurements [21].

#### Dependent variable: childhood overweight or obesity

We used two steps to process the variable. Initially, childhood overweight and obesity at age 36 months, 66 months, and 8 years were defined as a body mass index (BMI) from the 85th to the 94.9th percentile and greater than the 95th percentile for age and sex, respectively, based on the definition from the Department of HPA, Ministry of Health Welfare in Taiwan [22]. Subsequently, the dependent variable was categorized as a dichotomous variable for the advanced analysis in this study: childhood overweight or obesity (coded as 1) and non-overweight or obesity (coded as 0).

#### Independent variable: childhood RWG

Childhood RWG was defined as an increase of more than 0.67 in weight-for-age z-score, a measurement widely used and accepted in the literature [6], and the z-score was calculated using TBCS data. Subsequently, we calculated the time intervals of childhood RWG every six months from birth to 24 months of age in four periods: from birth to 6 months (birth-6 mo), from 6 months to 12 months (6 mo-12 mo), from 12 months to 18 months (12 mo-18 mo), and from 18 months to 24 months (18 mo-24 mo).

#### Potential covariates

Various factors were considered to be important for the occurrence of RWG and the development of overweight and obesity. We identified and classified potential covariates into three parts. The first part was related to prenatal influences, including gestational age, delivery method, and maternal smoking during pregnancy [23]. Gestational age was recorded from the National Birth Report Database, and the delivery method and maternal smoking during pregnancy were documented from the TBCS questionnaire completed at the age of 6 months.

The second part was breastfeeding duration [23, 24], which was documented from the survey questionnaire completed at the age of 18 months and was defined as partial breastfeeding until 12 months of age according to mothers’ responses. The third part was parental sociodemographic characteristics [24], including residential area, maternal nationality, and maternal educational achievement measured by the survey questionnaire completed at the age of 6 months and family income measured by the questionnaire completed during each wave of survey.

### Statistical analyses

We analysed the data in three steps. First, descriptive analyses of the distribution of childhood RWG and overweight or obesity are presented as frequencies and percentages, respectively. Specifically, the distribution of childhood RWG recorded the occurrence of RWG at each time interval and was categorized into several groups. For instance, some children’s RWG might have occurred in the period of birth-6 mo only, which was categorized into one group. Others might have begun in the period of 6 mo-12 mo and continued in the period of 12 mo-18 mo, which was categorized into another group.

Next, Pearson’s chi-squared (χ^2^) tests were used to examine the associations of childhood RWG with the potential determinants. In this process, childhood RWG was classified as a binary variable: children who had ever experienced RWG at any time interval before age 24 months and children who had never experienced RWG before age 24 months. Finally, logistic regression models with multiple covariates were used to obtain the adjusted odds ratio while controlling for covariates. We interpreted the coefficients to quantify the relationship between childhood RWG and overweight or obesity at 36 months, 66 months, and 8 years of age.

## Results

Table 1 presents the sociodemographic characteristics of the children. Of the 17,002 children, 52.6% were boys, and more than 90% of infants presented a normal birth weight, full-term birth, and singleton pregnancies. Moreover, 33.1% of the subjects were born via caesarean section (CS), and 5.7% were children of mothers who smoked during pregnancy. After birth, 12.9% of the children were partially breastfed until at least 12 months. Most mothers were native Taiwanese (88.2%), and 47.9% of the mothers had more than 15 years of education. Table 1 also demonstrated that children with low birthweight (47.9%), preterm birth (52.9%), and multiple parity birth (51.4%) were significantly associated with at least one occurrence of RWG.

**Table 1:** The distribution of children’s and parents’ characteristics and the association with rapid weight gain

Variables	Total		Ever RWG		No RWG		χ^2^	
	n	%	n	%	n	%		
Total	17,002	100.0	7509	44.2	9493	55.8		
Sex							0.03	
Boys	8948	52.6	3946	44.1	5002	55.9		
Girls	8054	47.4	3563	44.2	4491	55.8		
Birth weight							6.99	**
< 2500 g	1135	6.7	544	47.9	591	52.1		
> =2500 g	15,867	93.3	6965	43.9	8902	56.1		
Gestational age							81.41	***
Preterm	1379	8.1	730	52.9	649	47.1		
37 weeks	2212	13.0	1083	49.0	1129	51.0		
38 weeks	4532	26.7	1965	43.4	2567	56.6		
> =39 weeks	8879	52.2	3731	42.0	5148	58.0		
Singleton or multiple births							10.11	**
Singleton	16,539	97.3	7271	44.0	9268	56.0		
Multiple	463	2.7	238	51.4	225	48.6		
Delivery methods							3.85	
Caesarean section	5622	33.1	2542	45.2	3080	54.8		
Vaginal delivery	11,360	66.9	4956	43.6	6404	56.4		
Maternal smoking during pregnancy							0.09	
Yes	961	5.7	429	44.6	532	55.4		
No	16,041	94.4	7080	44.1	8961	55.9		
Breastfeeding until at least 12 months							19.81	***
Yes	2186	12.9	869	39.8	1317	60.3		
No	14,816	87.1	6640	44.8	8176	55.2		
Maternal nationality							8.15	**
Taiwanese	14,995	88.2	6683	44.6	8312	55.4		
Others	2005	11.8	826	41.2	1179	58.8		
Residential area							1.54	
Rural township	4716	27.7	2107	44.7	2609	55.3		
Urban township	7575	44.6	3306	43.6	4269	56.4		
City	4711	27.7	2096	44.5	2615	55.5		
Maternal education							8.82	*
< 9 years	2189	12.9	917	41.9	1272	58.1		
9–14 years	6661	39.2	2910	43.7	3751	56.3		
> =15 years	8126	47.9	3674	45.2	4452	54.8		
Family monthly income (NTD^†^)							3.49	
< 30,000	1829	10.8	774	42.3	1055	57.7		
30,000-49,999	5019	29.6	2204	43.9	2815	56.1		
50,000-69,999	4481	26.4	2005	44.7	2476	55.3		
> =70,000	5632	33.2	2505	44.5	3127	55.5		

* *p* < 0.05, ** *p* < 0.01, *** *p* < 0.001

†New Taiwan Dollars

### Distributions of RWG in children during all observational periods

Table 2 shows the distribution of childhood RWG from birth to age 24 months. In general, before age 24 months, 55.8% of children never experienced RWG, while 44.2% had at least one occurrence of RWG. Moreover, among children with at least one experience of RWG, 82.7% of children experienced only one period of RWG at birth- 6 mo (39.6%), 6 mo-12 mo (25.4%), 12 mo-18 mo (13.6%), and 18 mo-24 mo (4.1%).

**Table 2:** Distribution of rapid weight gain (RWG) before the age of two

Children’s experienced RWG	Total		Ever RWG
	n	%	%
Total	17,002	100.0	
No-RWG	9493	55.8	
Ever RWG	7509	44.2	100.0
At a single time interval			
Birth - 6 mo^*^	2975	17.5	39.6
6 mo - 12 mo	1911	11.2	25.4
12 mo - 18 mo	1024	6.0	13.6
18 mo - 24 mo	310	1.8	4.1
At other time intervals	1289	7.6	17.2

^*^Months of age

### The prevalence of childhood overweight or obesity according to RWG

**Fig. 1:**
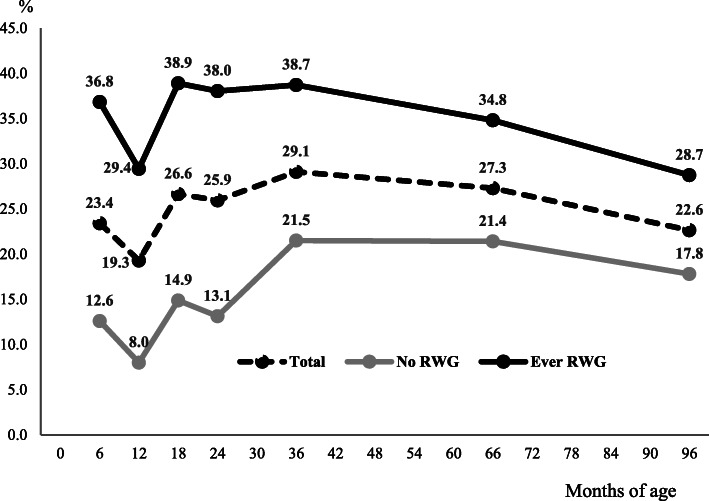
Prevalence of childhood overweight or obesity and rapid weight gain (RWG)

As Fig. 1 shows, the prevalence of childhood overweight or obesity among all children at 36 months, 66 months, and 8 years of age was 29.1, 27.3, and 22.6%, respectively. Moreover, the prevalence of children with at least one occurrence of RWG was approximately 30%, which was higher than that of children who did not experience RWG before the age of 24 months.

### Multivariable logistic regression of RWG and overweight or obesity

**Fig. 2:**
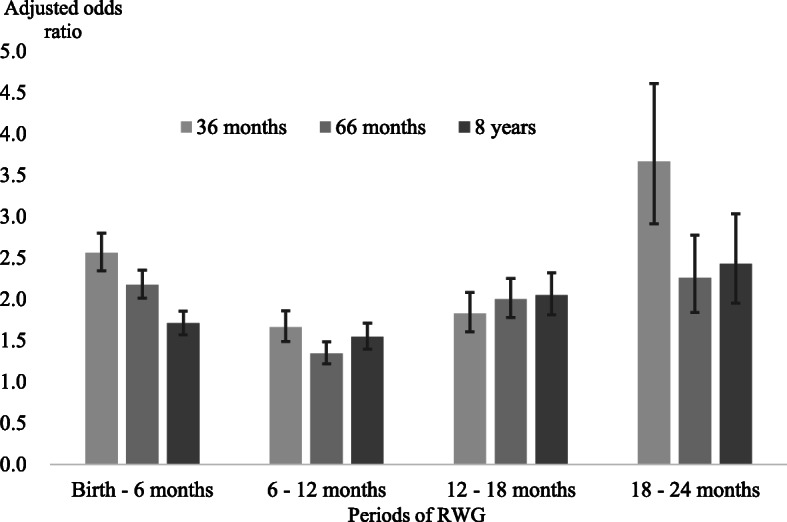
Adjusted odds ratio (including 95% confidence intervals, CIs) from the multiple logistic regression of childhood overweight or obesity at 36 months, 66 months, and 8 years of age according to each period of rapid weight gain (RWG) before the age of two

Figure 2 (or Appendix Table 1) illustrates the adjusted odds ratio (AOR) for the overweight or obesity predictions at 36 months, 66 months, and 8 years of age after controlling for potential covariates. In general, children who experienced RWG before age 24 months were more likely to be overweight or obese at age 36 months as well as at age 66 months and age 8 years. Furthermore, children with RWG at 18 mo-24 mo were more likely to become overweight or obese than other children without RWG with an AOR above 2 (age 36 months: AOR = 3.7, 95% CI = 2.9–4.6, 66 months: AOR = 2.3, 95% CI = 1.8–2.8, 8 years: AOR = 2.4, 95% CI = 2.0–3.0).

In addition, we tried to employ ordinal logistic regression separating the overweight and obese categories, and found that the results were almost no difference in findings using the dichotomous variable (Appendix Table 2). Thus, we went with the most parsimonious model in the current study.

## Discussion

This study, which analysed a representative longitudinal sample of children born in 2005, illustrates two findings. First, 17.5% of children experienced RWG in the first six months of life, compared to only 1.8% of children from 18-24 months of age. Second, children who experienced RWG had a significantly higher risk of overweight or obesity at preschool and school age, regardless of the occurrence of RWG at any time interval before the age of two.

Our results indicated that children’s growth before the age of two is important for physical health, as height and weight increase rapidly during this period [25]. Moreover, the results illustrated that the occurrences of RWG were widely distributed and decreased as children grew older. Identifying at a single and precise time interval of RWG for prevention of subsequent overweight or obesity may be difficult. Thus, it would be worthwhile to increase parental and health care provider awareness about preventing RWG during the first two years of a child’s life and not just focus on the specific timing of RWG. For instance, a set of well-child care visits was implemented as a strategy to screen and assess the growth and development of children after birth [26].

### The positive association between RWG in children and childhood overweight or obesity

Our findings are in line with those of earlier studies showing a connection between RWG in early life and subsequent overweight and obesity [7, 8]. Notably, the time interval of RWG occurrences would have different sensitivities for predicting overweight or obesity later in life. Therefore, rather than focusing on a single interval or the specific timing of RWG, childhood overweight and obesity surveillance using RWG screening should be continuous after birth or sustained at least until age 12 months.

Furthermore, we also suggest that potential factors should be considered for the prevention childhood RWG. First, children with premature births, a low birth weight or a younger gestational age may exhibit ‘catch-up growth’, and care should be given to avoid overweight and obesity or other chronic diseases later in life [27, 28]. Second, compared with milk formula feeding, children who may consume less energy and protein through breastfeeding were consistently less likely to experience RWG [29, 30]. Therefore, policies should encourage mothers to breastfeed exclusively, specifically mothers with lower education levels [31].

### Strengths and limitations

The collected data included indicators of birth outcomes, social environments, and lifestyles. Thus, the present study was able to clarify the association between RWG and overweight or obesity after controlling for other risk factors better than previous studies. The current study also has some limitations. First, the anthropometric data before the age of 8 years, which were documented from the Children’s Health Booklets, may have contained inaccuracies, and the primary caregiver reports after the age of 8 years were obtained from routine school health check-ups. However, earlier research has found that routine health checkup data relating to growth can be accurate [32]. Second, our findings should be generalized to the general population with caution, even though the TBCS was a large-scale study, employed random sampling, and recruited a homogeneous group of participants in terms of race/ethnicity.

## Conclusions

The current study using the panel data from a single nationally representative cohort in Taiwan found that childhood RWG increased the risk of subsequent overweight or obesity, regardless of the specific time interval during which RWG occurred before the age of two. Therefore, our findings reinforce the importance of monitoring childhood RWG continuously and show the risks of childhood RWG with respect to the development of overweight or obesity at preschool and school ages.

## Supplementary information

**Additional file 1.** Results of logistic regression model. **Table 1**. Multiple logistic regression of childhood overweight or obesity at 36 months, 66 months, and 8 years of age according to the period of rapid weight gain (RWG) before the age of two. **Table 2**. Ordinal logistic regression of childhood overweight or obesity at 36 months, 66 months, and 8 years of age according to the period of rapid weight gain (RWG) before the age of two.

## Data Availability

The datasets generated and analyzed during the current study are not publicly available due to the terms of consent to which the participants agreed, but data are however available upon reasonable request and with permission of the Health Promotion Administration at the Ministry of Health and Welfare in Taiwan.

## Abbreviations

NCDs: Non-Communicable Diseases
RWG: Rapid Weight Gain
AAP: The American Academy of Pediatrics
HPA: The Health Promotion Administration, Ministry of Health and Welfare
BMI: Body Mass Index
AOR: Adjusted Odds Ratio
