# Supplementary material for: Timing of rapid weight gain and its effect on subsequent overweight or obesity in childhood: findings from a longitudinal birth cohort study
Source: BMC Pediatr. 2020 Jun 12;20:293. doi: 10.1186/s12887-020-02184-9 (PMC7291582; doi:10.1186/s12887-020-02184-9)
Supplement: Supplementary file 1 — Additional file 1. Results of logistic regression model. Table 1. Multiple logistic regression of childhood overweight or obesity at 36 months, 66 months, and 8 years of age according to the period of rapid weight gain (RWG) before the age of two. Table 2. Ordinal logistic regression of childhood overweight or obesity at 36 months, 66 months, and 8 years of age according to the period of rapid weight gain (RWG) before the age of two. [file 12887_2020_2184_MOESM1_ESM.docx]

Appendixes

Table 1. Multiple logistic regression of childhood overweight and obesity at 36 months, 66 months, and 8 years of age according to the period of rapid weight gain (RWG) before the age of two.

| Rapid weight gain^†^ | | 36 months | | |  | 66 months | | |  | 8 years | | |
| --- | --- | --- | --- | --- | --- | --- | --- | --- | --- | --- | --- | --- |
|  |  | Adjusted OR^§^ | 95% CI^¶^ | |  | Adjusted OR | 95% CI | |  | Adjusted OR | 95% CI | |
|  |  |  | Lower | Upper |  |  | Lower | Upper |  |  | Lower | Upper |
| Model 1 | Birth - 6 months | 2.56 | 2.34 | 2.80 |  | 2.17 | 2.01 | 2.35 |  | 1.71 | 1.57 | 1.85 |
| Model 2 | 6 months - 12 months | 1.66 | 1.49 | 1.86 |  | 1.34 | 1.22 | 1.48 |  | 1.55 | 1.39 | 1.71 |
| Model 3 | 12 months - 18 months | 1.82 | 1.60 | 2.08 |  | 2.00 | 1.78 | 2.25 |  | 2.05 | 1.81 | 2.32 |
| Model 4 | 18 months - 24 months | 3.66 | 2.91 | 4.61 |  | 2.26 | 1.84 | 2.77 |  | 2.43 | 1.95 | 3.03 |

† After controlling for the demographic characteristics, social environment and breastfeeding.

§ OR: Odds Ratio; ¶ CI: Confidence Interval.

Table 2. Ordinal logistic regression of childhood overweight and obesity at 36 months, 66 months, and 8 years of age according to the period of rapid weight gain (RWG) before the age of two.

| Rapid weight gain^†^ | | 36 months | | |  | 66 months | | |  | 8 years | | |
| --- | --- | --- | --- | --- | --- | --- | --- | --- | --- | --- | --- | --- |
|  |  | Adjusted OR^§^ | 95% CI^¶^ | |  | Adjusted OR | 95% CI | |  | Adjusted OR | 95% CI | |
|  |  |  | Lower | Upper |  |  | Lower | Upper |  |  | Lower | Upper |
| Model 1 | Birth - 6 months | 2.56 | 2.35 | 2.80 |  | 2.05 | 1.86 | 2.26 |  | 1.68 | 1.52 | 1.86 |
| Model 2 | 6 months - 12 months | 1.70 | 1.52 | 1.89 |  | 1.38 | 1.23 | 1.56 |  | 1.52 | 1.34 | 1.72 |
| Model 3 | 12 months - 18 months | 1.92 | 1.69 | 2.18 |  | 2.13 | 1.85 | 2.46 |  | 2.12 | 1.83 | 2.46 |
| Model 4 | 18 months - 24 months | 3.74 | 3.00 | 4.66 |  | 2.29 | 1.78 | 2.94 |  | 2.18 | 1.67 | 2.85 |

† After controlling for the demographic characteristics, social environment and breastfeeding.

§ OR: Odds Ratio; ¶ CI: Confidence Interval.
